# Preventive treatment patterns and treatment satisfaction in migraine: results of the OVERCOME (EU) study

**DOI:** 10.1186/s10194-023-01623-z

**Published:** 2023-07-17

**Authors:** Julio Pascual, Tommaso Panni, Grazia Dell’Agnello, Saygin Gonderten, Diego Novick, Stefan Evers

**Affiliations:** 1 Hospital Universitario Marqués de Valdecilla, Universidad de Cantabria and IDVAL, Santander, Spain; 2 Eli Lilly Deutschland GmbH, Bad Homburg, Germany; 3 Eli Lilly Italia SpA, Sesto Fiorentino, Italy; 4 Eli Lilly and Company Ltd, Dubai, UAE; 5 Eli Lilly and Company Ltd, Bracknell, UK; 6 University of Münster, Münster, Germany; 7 Department of Neurology, Lindenbrunn Hospital, Coppenbrügge, Germany

**Keywords:** OVERCOME (EU), Migraine, Severe headache, Preventive medication, Treatment patterns, Treatment satisfaction

## Abstract

**Background:**

Insights into the burden, needs and treatment of migraine from internet-based surveys in diverse real-world migraine populations are needed, especially at a time when novel preventive migraine medications are becoming part of the therapeutic armamentarium. The objectives of this analysis are to describe traditional preventive (orals and onabotulinum toxin A) treatment patterns in the OVERCOME (EU) study migraine cohort, as well as treatment patterns and patient satisfaction with current treatment in a subgroup of respondents eligible for migraine preventive medication.

**Methods:**

The cross-sectional non-interventional OVERCOME (EU) study was conducted (October 2020–February 2021) via an online survey among adults (aged ≥ 18 years) resident in Germany or Spain. Participants, registered in existing online panels, who were willing to provide consent were considered. The migraine cohort included participants reporting headache/migraine in the past year, identified based on a validated migraine diagnostic questionnaire and/or self-reported physician diagnosis. A subgroup of survey respondents defined as eligible for migraine preventive medication at the point in time the cross-sectional survey was taken was also analysed. Variables assessed included sociodemographic and migraine-related clinical characteristics, preventive (traditional and calcitonin gene-related peptide monoclonal antibodies) treatment patterns and patient satisfaction with current treatment. Results are descriptive only.

**Results:**

Of the 20,756 participants in the migraine cohort, 78.5% sought professional medical care, 50.8% received a migraine diagnosis and only 17.7% had ever used preventive medication. Half (53.3%) of participants currently using preventives took their most recent medication for six months or less. Most patients (73.9%) classified as eligible for preventive medication (based on headache frequency and/or at least moderate disability due to migraine) reported not using traditional preventives and many of those who did (66.8%) were not satisfied with their current standard of care.

**Conclusions:**

Our findings highlight the low proportion of people diagnosed with migraine despite a higher rate of consultation and suggest the need for better access to treatment for people with migraine and new preventive therapies with improved efficacy and safety profiles to improve adherence and patient satisfaction.

## Background

Migraine is a debilitating neurological disease with an estimated overall prevalence of 15% in Europe [1]. According to Kantar’s 2017 National Health and Wellness Survey, 21% of adult respondents at least 18 years of age in the EU5 (France, Germany, Italy, Spain and the United Kingdom) reported experiencing migraine, with only 10% self-reporting a physician’s diagnosis of migraine [2]. These data further support evidence from the cross-sectional, questionnaire-based Eurolight survey in ten European countries that many people with migraine do not seek professional medical care [3].

With new preventive migraine medications as therapeutic options, in addition to the anticipated approval of new acute treatments, it is important to understand the epidemiology and burden of migraine. Furthermore, it is necessary to identify barriers to the initiation of preventive and acute migraine therapies and understand how the introduction of new classes of migraine medication influences healthcare delivery and migraine care. However, insights into the burden, needs and treatment of migraine within diverse real-world migraine populations are not or are only partially available from medical databases (e.g., clinical trials or registries) because of reluctance on the part of people with migraine to consult physicians. Therefore, an alternative approach to gathering this information is required.

Unfortunately, several prior population-based surveys of migraine were limited by their geographic location or population subset and/or may not reflect current treatment patterns [4–6]. However, real-world studies using internet-based surveys, such as the ObserVational survey of the Epidemiology, tReatment and Care Of MigrainE (Europe) (OVERCOME [EU]) conducted in Germany and Spain – part of an overarching study programme that also includes the United States (US) and Japan [7–9] – allow access to a broad population of people with migraine, irrespective of whether they have been diagnosed with migraine by a physician and/or are seeking medical care. This internet-based survey approach allows for large numbers of study participants, involves people fulfilling internationally recognised migraine classification criteria or with a self-reported physician diagnosis of migraine, or both, and facilitates the collection of data on treatment satisfaction and the behaviour of people with migraine in real life.

The objectives of this analysis are to describe the sociodemographic and migraine-related clinical characteristics of the OVERCOME (EU) study migraine cohort, as well as traditional preventive treatment patterns (i.e., for antidepressants, antihypertensives, antiseizures and onabotulinum toxin A but excluding calcitonin gene-related peptide [CGRP]-monoclonal antibodies [mAbs]) in this cohort and in the subgroup of survey respondents defined as eligible for migraine preventive medication at the point in time the cross-sectional survey was taken. We also report on patient satisfaction with current treatment in survey respondents eligible for migraine preventive medication.

## Methods

### Design and setting

Data were obtained from a non-interventional, cross-sectional, observational study conducted via an online survey between October 2020 and February 2021 among adults resident in Germany and Spain. Participants registered in existing opt-in online survey panels (Kantar Profiles [Lightspeed] global panel and its partners) were invited to participate in the health survey without prior knowledge of the specific health topic.

**Fig. 1:**
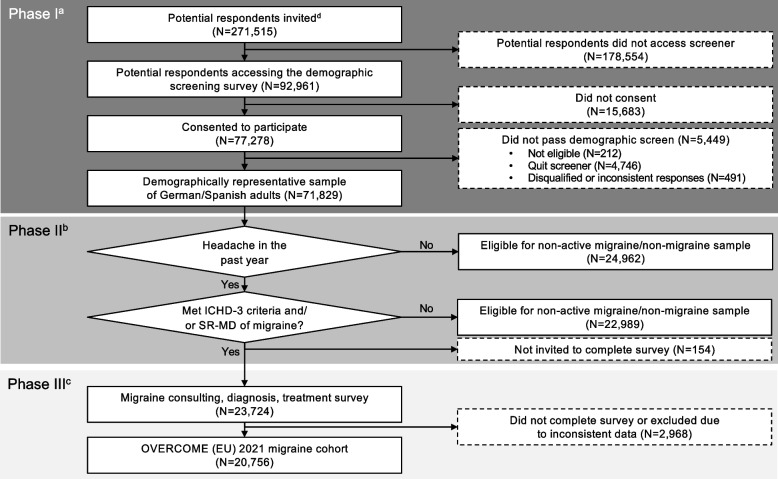
Consort diagram for OVERCOME (EU) migraine cohort. Consort diagram for OVERCOME (EU) 2021 migraine cohort (*N* = 20,756). ^a^Phase I = creating a demographically representative sample of German/Spanish adults. ^b^Phase II = identifying respondents with migraine. ^c^Phase III = establishing the migraine cohort. ^d^Targeted sampling to represent the German/Spanish adult population in terms of key demographic characteristics (age and sex) was applied. Abbreviations: ICHD-3, International Classification of Headache Disorders, 3^rd^ edition; OVERCOME (EU), ObserVational survey of the Epidemiology, tReatment and Care Of MigrainE (Europe); SR-MD, self-reported medical diagnosis of migraine

A three-phase approach was taken to establishing the migraine cohort. In phase I, a sample population that was demographically representative of the German/Spanish population was created via quota sampling (Fig. 1). Sample performance was monitored daily, based on pre-specified demographics (age and sex), to ensure the representativeness of the data and the random selection process was refined to target panel members matching demographic characteristics for quotas not yet reached. Inclusion criteria for phase I were (a) aged 18 years or older, (b) resident in Germany or Spain and able to read and write Spanish or German, and (c) online survey panel member with internet access and ability to provide electronic informed consent.

**Fig. 2:**
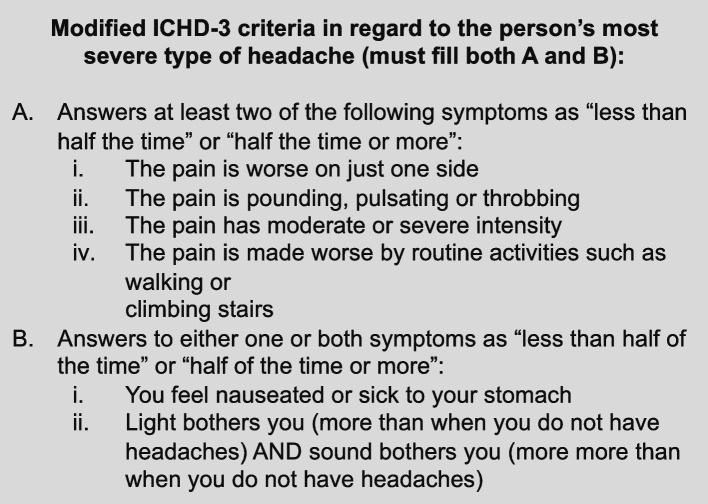
Modified International Classification of Headache Disorders, 3^rd^ edition screening criteria [10]. Abbreviations: ICHD-3, International Classification of Headache Disorders, 3^rd^ edition

In phase II, respondents with migraine were identified in the demographically representative population. Respondents were asked a series of questions around health and comorbidities, including whether they had at least one headache in the past 12 months not associated with head injury, illness or hangover. Of these potentially eligible individuals, individual respondents were then identified as having migraine, either by a self-reported physician diagnosis of migraine or fulfilling International Classification of Headache Disorders, 3^rd^ edition (ICHD-3) criteria [10] (Fig. 2), or both.

A migraine cohort was established in phase III. To allow for analyses of smaller subgroups, the planned sample size of the migraine cohort was 20,000 participants in total (10,000 each in Germany and Spain). Respondents included in the migraine cohort were required to answer all survey questions assessing the consultation, treatment and impact of migraine.

### Survey instrument

The validated English language survey was translated to each local language using a three-step process involving three independent healthcare experienced linguists. The approximate length of time taken to complete the OVERCOME (EU) survey was 30 min for respondents in the migraine cohort. The main categories of questions included in the survey are outlined in Table 1. The non-migraine (control) cohort survey was approximately five minutes in length and was limited to questions about demographics, comorbidities, HCRU and attitudes/perceptions about people with migraine.

**Table 1:** Main categories of questions included in the OVERCOME (EU) survey

**Category**	**Question topics**
Socioeconomic status	Family circumstances; work situation; educational level; income
Lifestyle and health status	(Cardiovascular) comorbidities; BMI; smoking; alcohol use; impact of COVID-19
Diagnosis and healthcare resource utilization	Self-reported; based on ICHD-3 criteria; age at first diagnosis; medical tests; diary use; frequency of HCP visits; time between making appointments and visits; insurance
Clinical features of migraine	Headache frequency; age at first attack; allodynia symptoms (ASC-12) [11]; aura, sensory, timing and duration of attacks; sleep interference; menstrual migraine
Use of medication	Former and current use of acute and/or preventive medication (prescription and/or OTC; on agent level); reasons for, order, frequency, timing and duration of use of medications; prescribing HCP/location; medication access issues; effectiveness of each medication (on agent level); reasons for delayed use, switching or stopping, or not taking medications; out-of-pocket payments for medications; preventive therapies except medications
Barriers to care	Hesitation to consult HCP; reasons for hesitation
Burden of migraine and QoL	Migraine disability (MIDAS) [12, 13]; migraine-specific QoL (MSQ v2.1) [14–16]; impact of migraine on different aspects of (daily) life (EQ-5D-5L) [17]; driving attitudes and behaviour; work productivity and activity impairment (WPAI) [18]; impact of migraine compared to other patients; impact of migraine on family (IMPAC scale) [19]; interictal burden (MIBS-4) [20, 21]; depression (PHQ-8) and anxiety (GAD-7) [22]
Stigma	Reputation; prejudices of others

*Abbreviations*: *ASC-12* Allodynia Symptom Checklist-12, *BMI* Body mass index, *COVID-19* Coronavirus disease 2019, *EQ-5D-5L* EQ-5D-5-Levels, *GAD-7* Generalized Anxiety Disorder scale 7, *HCP* Healthcare professional, *ICHD-3* The International Classification of Headache Disorders, 3^rd^ edition, I*MPAC* Impact of Migraine on Partners and Adolescent Children scale, *MIBS-4* Migraine Interictal Burden Scale-4, *MIDAS* Migraine Disability Assessment, *MSQ v2.1* Migraine-Specific Quality-of-Life Questionnaire version 2.1, *OTC* Over-the-counter, *PHQ-8* Patient Health Questionnaire-8, *QoL* Quality of life; *WPAI* Work Productivity and Activity Impairment questionnaire

Migraine-related clinical characteristics of the migraine cohort are reported in this analysis using three of the patient-reported outcome measures included in the OVERCOME (EU) survey (Table 1): the Migraine Disability Assessment (MIDAS), the Migraine Interictal Burden Scale-4 (MIBS-4) and the Migraine-Specific Quality-of-Life Questionnaire version 2.1 (MSQ v2.1). MIDAS assesses migraine-related disability, quantifying the number of days a person has missed or had reduced productivity at work, home or in social settings over the past three months and assigning disability grades based on the numbers of days, with higher scores indicating more severe disability [12, 13]. MIBS-4 measures the burden related to headache in the time between attacks, specifically disruption at work and school, diminished family and social life, difficulty planning and emotional difficulty over the previous four weeks on days without a headache attack [20, 21]. MSQ v2.1 is a self-administered health status instrument developed to address physical and emotional limitations of specific concern to individuals suffering from migraine headaches across three domains: (1) role function – restrictive, (2) role function – preventive and (3) role function – emotional function [14–16].

### Statistical analyses

This analysis describes the sociodemographic and migraine-related clinical characteristics of the OVERCOME (EU) study migraine cohort, overall and by headache days/month (HD/m) subgroup (0–3, 4–7, 8–14 and ≥ 15 HD/m). Preventive treatment patterns are then described for the migraine cohort before focussing specifically on traditional preventive treatment patterns (i.e., for antidepressants, antihypertensives, antiseizures and onabotulinum toxin A but excluding CGRP-mAbs). CGRP-mAb users were excluded from these analyses of treatment patterns to identify unmet needs within this patient population, which may require newer preventive migraine medications with different modes of action to address. In order to take into account the large proportion of respondents in the 0–3 HD/m subgroup, traditional preventive treatment patterns (all excluding CGRP-mAb users) are also described for the subgroup of survey respondents from the migraine cohort specifically eligible for migraine preventive medication at the point in time the cross-sectional survey was taken; eligible respondents were defined as medically diagnosed patients, with a mean ≥ 4 migraine HD/m over the last 90 days and MIDAS score ≥ 11 [23]. Finally, this analysis also describes patient satisfaction with current treatment in the subgroup eligible for migraine preventive medication, excluding CGRP-mAb users.

The OVERCOME (EU) study sample size was based on the sample size of the sister study (OVERCOME [US]) [7] and on the key study objectives. The overall migraine cohort and specific subgroups included in this analysis were analysed using descriptive statistics. Continuous variables are reported as means with standard deviations (SDs), or medians and ranges, as appropriate. Categorical variables are summarised as frequencies and percentages. SAS version 9.4 software was used to undertake all analyses.

## Results

The OVERCOME (EU) 2021 migraine cohort was comprised of 20,756 respondents in Germany and Spain (Fig. 1).

### Sociodemographic and migraine-related clinical characteristics

The sociodemographic and migraine-related clinical characteristics of the OVERCOME (EU) migraine cohort, overall and by HD/m subgroup, as related to the aforementioned objectives, are described in detail in Table 2. Individuals in the migraine cohort had a mean age of 40.5 years and 60.3% were female, with the proportion of females rising in subgroups with increasing HD/m (range 57.0–72.6%). The majority of respondents (65.6%) were married or living with a partner, and most were employed full or part time (70.4%); however, the number of respondents in employment decreased in the higher HD/m subgroups (58.2% in ≥ 15 HD/m subgroup). The mean (SD) age at migraine diagnosis, among those respondents with a migraine diagnosis (57.6%), was 24.2 (10.8) years.

**Table 2:** Sociodemographic and migraine-related clinical characteristics in the OVERCOME (EU) migraine cohort (*N* = 20,756)

**Migraine cohort**	**0–3 HD/m** **(*n* = 13,759)**	**4–7 HD/m** **(*n* = 4203)**	**8–14 HD/m** **(*n* = 1730)**	** ≥ 15 HD/m** **(*n* = 1064)**	**Total** **(*N* = 20,756)**
**Age (years), mean (SD)**	40.1 (13.5)	40.7 (13.3)	41.2 (13.5)	42.2 (13.9)	40.4 (13.5)
**Sex (female), n (%)**	7846 (57.0)	2717 (64.6)	1177 (68.0)	772 (72.6)	12,512 (60.3)
**Marital status, n (%)**					
** Married or living with partner**	8972 (65.2)	2831 (67.4)	1144 (66.1)	664 (62.4)	13,611 (65.6)
** Single, separated, divorced or widowed**	4697 (34.1)	1340 (31.9)	580 (33.5)	393 (36.9)	7010 (33.8)
** Prefer not to answer**	90 (0.7)	32 (0.8)	6 (0.3)	7 (0.7)	135 (0.7)
**Employment status, n (%)**					
** Employed full or part time**	9815 (71.3)	3023 (71.9)	1163 (67.2)	619 (58.2)	14,620 (70.4)
** Not employed**^a^	3834 (27.9)	1147 (27.3)	557 (32.2)	429 (40.2)	5967 (28.7)
** Prefer not to answer**	110 (0.8)	33 (0.8)	10 (0.6)	16 (1.5)	169 (0.8)
**Previously diagnosed with migraine by healthcare provider, n (%)**	7269 (52.8)	2805 (66.7)	1169 (67.6)	705 (66.3)	11,948 (57.6)
**Age at migraine diagnosis,**^b^** mean (SD)**	24.0 (10.6)	24.4 (11.0)	25.1 (11.0)	24.3 (11.6)	24.2 (10.8)
**HD/m, mean (SD)**	1.8 (1.0)	5.4 (1.0)	10.3 (1.7)	20.8 (4.8)	4.2 (4.9)
**Number of comorbidities (excluding migraine), n (%)**					
**1**	3667 (26.7)	866 (20.6)	326 (18.8)	156 (14.7)	5015 (24.2)
**2**	2716 (19.7)	772 (18.4)	324 (18.7)	145 (13.6)	3957 (19.1)
**3 + **	4305 (31.3)	1868 (44.4)	881 (50.9)	666 (62.6)	7720 (37.2)
**MIDAS grade,**^c^** n (%)**					
**I – little or no disability**	6689 (48.6)	1043 (24.8)	291 (16.8)	154 (14.5)	8177 (39.4)
**II – mild disability**	2988 (21.7)	728 (17.3)	180 (10.4)	80 (7.5)	3976 (19.2)
**III – moderate disability**	2438 (17.7)	1036 (24.6)	382 (22.1)	148 (13.9)	4004 (19.3)
**IV – severe disability**	1644 (11.9)	1396 (33.2)	877 (50.7)	682 (64.1)	4599 (22.2)
**MIBS-4 total score,**^d^** n (%)**					
**0 – no interictal burden**	4223 (30.7)	919 (21.9)	345 (19.9)	159 (14.9)	5646 (27.2)
**1–2 – mild interictal burden**	2156 (15.7)	642 (15.3)	242 (14.0)	143 (13.4)	3183 (15.3)
**3–4 moderate interictal burden**	1714 (12.5)	543 (12.9)	213 (12.3)	147 (13.8)	2617 (12.6)
**5 + – severe interictal burden**	5666 (41.2)	2099 (49.9)	930 (53.8)	615 (57.8)	9310 (44.9)
**MSQ v2.1 score,**^e^** mean (SD)**					
**Role function – restrictive**	67.2 (21.3)	56.6 (19.2)	53.1 (19.6)	47.5 (21.5)	62.9 (21.8)
**Role function – preventive**	74.9 (22.9)	67.8 (22.9)	64.8 (23.5)	60.9 (25.5)	71.9 (23.5)
**Role function – emotional function**	74.6 (24.1)	65.4 (24.5)	61.2 (25.4)	55.1 (27.3)	70.6 (25.2)

*Abbreviations*: *HD/m* Headache days per month, *MIBS-4* Migraine Interictal Burden Scale-4, *MIDAS* Migraine Disability Assessment, *MSQ v2.1* Migraine-Specific Quality-of-Life Questionnaire version 2.1, *SD* Standard deviation

^a^Not employed includes not employed and looking for work; not employed and not looking for work; long- or short-term disability; student; homemaker; and retired

^b^Among those previously diagnosed with migraine by healthcare provider. *n* = 5710, 2245, 938 and 575 for 0–3, 4–7, 8–14 and ≥ 15 HD/m, respectively, and *n* = 9468 for total migraine cohort. Note: age at migraine diagnosis was not recorded for all participants who were previously diagnosed with migraine by a healthcare provider

^c^MIDAS quantifies the number of days a person has missed or had reduced productivity at work, home or social settings over the past three months. Disability grades are then assigned based on the numbers of days, with higher scores indicating more severe disability: grade I = little or no disability (MIDAS score 0–5); grade II = mild (score 6–10); grade III = moderate (score 11–20); and grade IV = severe (score ≥ 21). The MIDAS instrument is considered reliable and valid and is correlated with clinical judgement regarding the need for medical care [5, 24]. Spanish and German versions of the MIDAS instrument are also validated [25, 26]

^d^MIBS-4 is a four-item instrument that measures the burden related to headache in the time between attacks [20, 21]. The self-administered instrument consists of four items that address disruption at work and school, diminished family and social life, difficulty planning and emotional difficulty. The questionnaire specifically asks about the effect of the disease over the past four weeks on days without a headache attack. Response options include: ‘don’t know/not applicable’, ‘never’, ‘rarely’, ‘some of the time’, ‘much of the time’ or ‘most or all of the time’. Each response has an associated numerical score, with the summation across all four items resulting in a total score ranging from 0 to 12, and the level of interictal burden being categorised into the following: 0 for none, 1–2 mild, 3–4 moderate, and > 5 severe

^e^MQS v2.1 is a self-administered health status instrument developed to address physical and emotional limitations of specific concern to individuals suffering from migraine headaches [14]. The instrument consists of 14 items that address three domains: (1) role function – restrictive, (2) role function – preventive and (3) role function – emotional function, using a 6-point Likert-type scale of ‘none of the time’, ‘a little bit of the time’, ‘some of the time’, ‘a good bit of the time’, ‘most of the time’ and ‘all of the time’. Raw scores for each dimension are computed as a sum of item responses, with the collective sum providing a total raw score that is then converted to a 0–100 scale, with higher scores indicating a better health-related quality of life (HRQoL), and a positive change in scores reflecting functional improvement [15, 16]

Approximately, 37% of individuals in the migraine cohort (37.2%) had three or more comorbidities, with the proportion rising in subgroups with increasing HD/m (range 31.3–62.6%) (Table 2). More individuals reported severe levels of disability, as measured by MIDAS grade, in the ≥ 15 HD/m subgroup (64.1% Grade IV) than in the 0–3 HD/m group (11.9%, respectively), as would be expected. Similarly, the proportion of participants reporting severe interictal burden, as indicated by a MIBS-4 total score of 5 + , was higher in the ≥ 15 HD/m subgroup (57.8%) than in the 0–3 HD/m group (41.2%). MSQ v2.1 scores for each of the three domains (role function – restrictive, preventive and emotional function) decreased across the subgroups as the number of HD/m increased.

### Preventive treatment patterns in OVERCOME (EU) migraine cohort

**Fig. 3:**
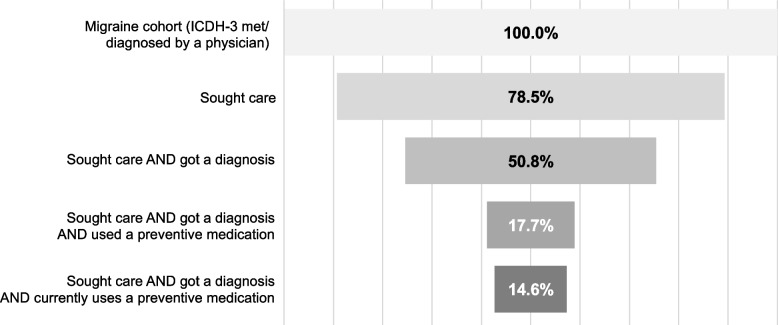
Overview of steps taken by participants in the OVERCOME (EU) migraine cohort (*N* = 20,756) to seek care and prevent migraine. Sought care = ever sought care from physician during lifetime for severe headache/migraine. Got a diagnosis = self-reported migraine diagnosed by physician. Used a preventive medication = ever used a preventive medication during lifetime for severe headache/migraine. Currently uses a preventive medication = has taken or used a preventive medication in the last three months. ICHD-3, International Classification of Headache Disorders, 3^rd^ edition

Of the 20,756 participants in the OVERCOME (EU) migraine cohort, 78.5% sought care from a physician for severe headache/migraine at some point in their lifetime; however, only 50.8% received a migraine diagnosis (Fig. 3). Of note, only 17.7% of those individuals who sought care and got a diagnosis reported ever using preventive medication for migraine and only 14.6% had used preventive medication within the last three months.

**Fig. 4:**
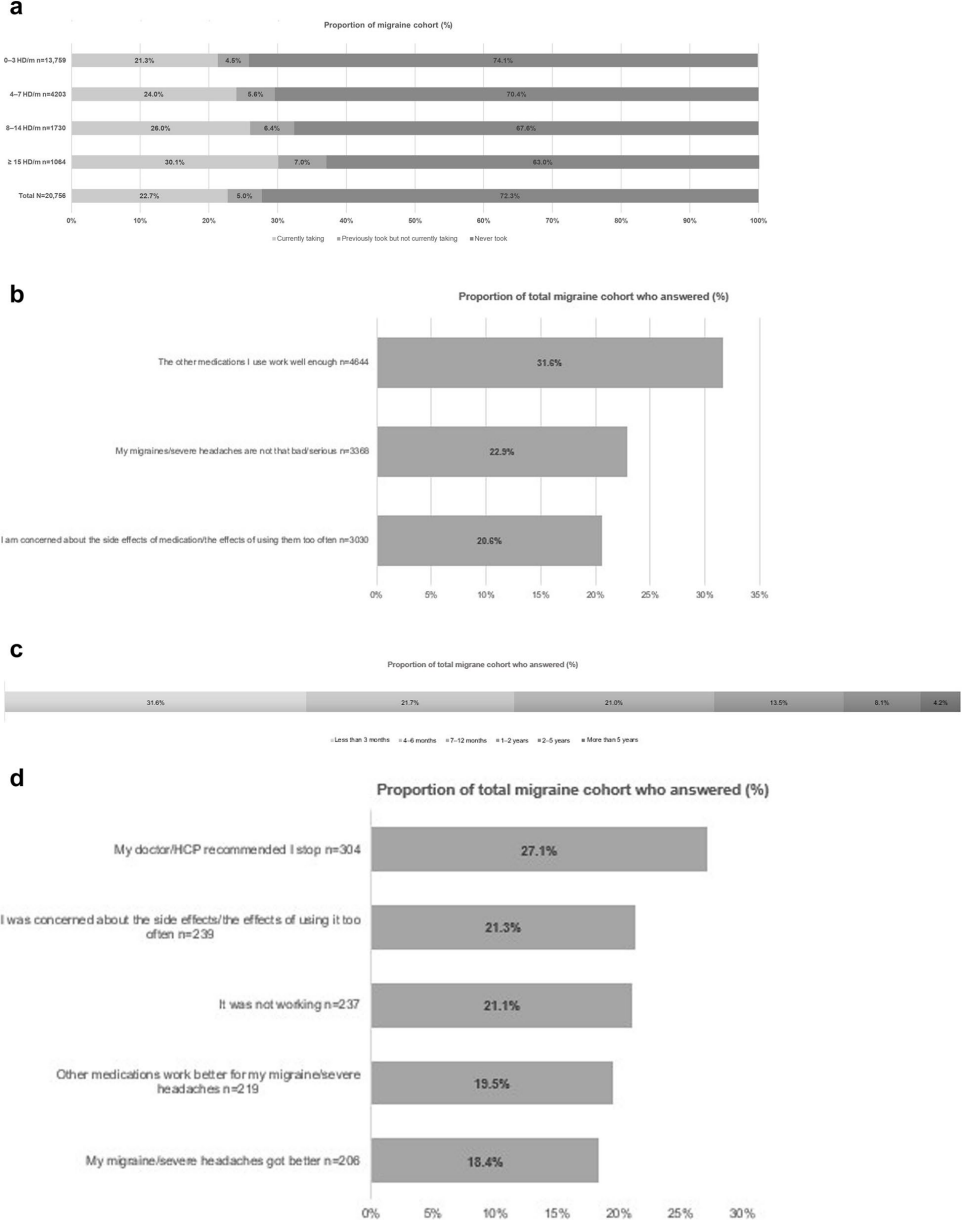
Preventive treatment patterns in the OVERCOME (EU) migraine cohort. **a** Experience with preventive medication^a^. **b** Top three reasons for never using preventive medication^b^ (*n* = 14,706). **c** Length of treatment of most recent preventive medication^c^ (excluding CGRP-mAbs; *n* = 1121). **d** Top five reasons for stopping preventive medication^d^ (excluding CGRP-mAbs; *n* = 1121). ^a^Question regarding experience with preventive medication was asked to all survey respondents. Preventive medication includes oral and injectable (monoclonal antibodies/botox) medications. ^b^Question regarding reasons for never using preventive medication was asked to respondents who had not been prescribed blood pressure/heart, antiseizure, antidepressant, or injectable (monoclonal antibodies/botox) medications ever for any reason OR had used such medication(s) for a health condition other than migraine/could not remember what the medication was used for. ^c^Question regarding length of treatment of most recent preventive medication (excluding monoclonal antibodies/botox) was asked to respondents who had used, and stopped using, blood pressure/heart, antiseizure or antidepressant medication(s) in the past to prevent or reduce the frequency, severity, or duration of migraine or severe headaches and specifically regarding the most recent one. Response options were based on a 6-point Likert scale (1 = Less than 3 months, 2 = 4 to 6 months, 3 = 7 to 12 months, 4 = 1 to 2 years, 5 = 2 to 5 years, 6 = More than 5 years). ^d^Question regarding reasons for stopping preventive medication (excluding monoclonal antibodies/botox) was asked to respondents who had used, and stopped using, blood pressure/heart, antiseizure or antidepressant medication(s) in the past to prevent or reduce the frequency, severity, or duration of migraine or severe headaches. HD, headache days

Overall, 72.3% of participants in the migraine cohort had never taken preventive medication, with the proportion of individuals falling in subgroups with increasing HD/m (from 74.1% to 63.0%) (Fig. 4A). The top three reasons for never taking preventative medication were efficacy of other medications (31.6%), migraines/severe headaches not being serious enough for treatment (22.9%) and concerns about side effects (20.6%) (Fig. 4B).

In the migraine cohort, 10.8% of participants reported currently using three or more traditional preventive medications (excluding CGRP-mAbs), while 4.2% used two preventives and 7.7% used one (Table 3). Antiseizure, antidepressant and antihypertensive medications were currently used by a similar proportion of participants in the migraine cohort (13.5, 14.5 and 15.1%, respectively), whereas onabotulinum toxin A (indicated specifically for chronic migraine) was used in the last three months by only 2.4% of survey respondents.

**Table 3:** Traditional preventive treatment patterns in OVERCOME (EU) migraine cohort and in survey respondents eligible for migraine preventive medication (excluding calcitonin gene-related peptide-monoclonal antibody users)

**OVERCOME (EU) migraine cohort**	**0–3 HD/m** **(*n* = 13,759)**	**4–7 HD/m** **(*n* = 4203)**	**8–14 HD/m** **(*n* = 1730)**	** ≥ 15 HD/m** **(*n* = 1064)**	**Total** **(*N* = 20,756)**
**Currently**^**b**^** using preventives,**^**c**^** n (%)**					
1 preventive	943 (6.9)	358 (8.5)	171 (9.9)	129 (12.1)	1601 (7.7)
2 preventives	514 (3.7)	188 (4.5)	92 (5.3)	68 (6.4)	862 (4.2)
≥ 3 preventives	1475 (10.7)	463 (11.0)	186 (10.8)	123 (11.6)	2247 (10.8)
**Current**^**b**^** preventive medication category,**^**c**^** n (%) – not mutually exclusive**					
Antiseizure^d^	1793 (13.0)	604 (14.4)	235 (13.6)	171 (16.1)	2803 (13.5)
Antidepressant^e^	1903 (13.8)	628 (14.9)	282 (16.3)	198 (18.6)	3011 (14.5)
Antihypertensive^f^	2011 (14.6)	649 (15.4)	284 (16.4)	180 (16.9)	3124 (15.1)
Onabotulinum toxin A	322 (2.3)	94 (2.2)	45 (2.6)	29 (2.7)	490 (2.4)
**Survey respondents eligible for migraine preventive medication**^**a**^	**N/A**	**4–7 HD/m** **(*n* = 1458)**	**8–14 HD/m** **(*n* = 792)**	** ≥ 15 HD/m** **(*n* = 499)**	**Total** **(*N* = 2749)**
**Currently**^**b**^** using preventives,**^**c**^** n (%)**					
1 preventive		173 (11.9)	97 (12.2)	79 (15.8)	349 (12.7)
2 preventives		73 (5.0)	50 (6.3)	41 (8.2)	164 (6.0)
≥ 3 preventives		104 (7.1)	59 (7.4)	41 (8.2)	204 (7.4)
**Current**^b^** preventive medication category,**^**c**^** n (%) – not mutually exclusive**					
Antiseizure^d^		170 (11.7)	99 (12.5)	72 (14.4)	341 (12.4)
Antidepressant^e^		201 (13.8)	115 (14.5)	99 (19.8)	415 (15.1)
Antihypertensive^f^		202 (13.9)	121 (15.3)	80 (16.0)	403 (14.7)
Onabotulinum toxin A		37 (2.5)	18 (2.3)	12 (2.4)	67 (2.4)

*CGRP-mAbs* calcitonin gene-related peptide-monoclonal antibodies, *HD/m* headache days per month, *MIDAS* Migraine Disability Assessment

^a^Patients with ≥ 4 HD/m on average over the last 90 days and ≥ 11 (MIDAS). Users of CGRP-mAbs are excluded

^b^Current defined as taken or used in the last three months for preventive medications

^c^Excluding CGRP-mAbs

^d^Antiseizure medications included valproic acid/valproate, gabapentin, pregabalin, topiramate, zonisamide, levetiracetam, clonazepam, lamotrigine, carbamazepine, oxcarbazepine and ‘other antiseizure medication’. Not all medications are licensed in both Germany and Spain

^e^Antidepressant medications included amitriptyline, desvenlafaxine, doxepin, escitalopram, fluoxetine, imipramine, nortriptyline, paroxetine, sertraline, venlafaxine, clomipramine, trazodone, mianserin, fluvoxamine, sulpiride, mirtazapine, citalopram, opipramol and ‘other antidepressant medication’. Not all medications are licensed in both Germany and Spain

^f^Antihypertensive medications included atenolol, candesartan, lisinopril, metoprolol, nifedipine, propranolol, verapamil, diltiazem, nicardipine, captopril, enalapril, telmisartan and ‘other blood pressure or heart medication’. Not all medications are licensed in both Germany and Spain

Interestingly, 53.3% of participants took their most recent preventive medication (excluding CGRP-mAbs) for six months or less (Fig. 4C). The top five reasons for stopping preventive medication were physician recommendation (27.1%), concerns about side effects (21.3%), lack of efficacy (21.1%), efficacy of other medications (19.5%) and improvement in migraine/severe headache (18.4%) (Fig. 4D).

### Preventive treatment patterns and treatment satisfaction in subgroup of survey respondents eligible for migraine preventive medication who had never used CGRP-mAbs

The subgroup of survey respondents eligible for migraine preventive medication at the point in time the cross-sectional survey was taken included 2,749 participants in Germany and Spain (13.2% of the overall migraine cohort). The mean (SD) age of this subgroup of individuals was 40.7 (12.9) years and 70.4% of respondents were female. The mean (SD) time to diagnosis in this subgroup of respondents was 2.8 (5.3) years and the mean (SD) number of migraine HD/m was 9.4 (6.1).

Most participants (73.9%) eligible for preventive medication did not report currently taking a preventive. A smaller proportion (7.4%) of the eligible subgroup took three or more preventives in the last three months compared with the overall migraine cohort (10.8%), whereas a larger proportion (12.7%) took one preventive (vs. 7.7% in the migraine cohort) (Table 3). The proportions of eligible participants taking one, two, or three or more preventives increased in subgroups with increasing HD/m. As reported for the overall migraine cohort, antiseizure, antidepressant and antihypertensive medications were currently used by a similar proportion of preventive eligible participants (12.4, 15.1 and 14.7%, respectively), whereas onabotulinum toxin A was used in the last three months by only 2.4% of preventive eligible respondents.

**Fig. 5:**
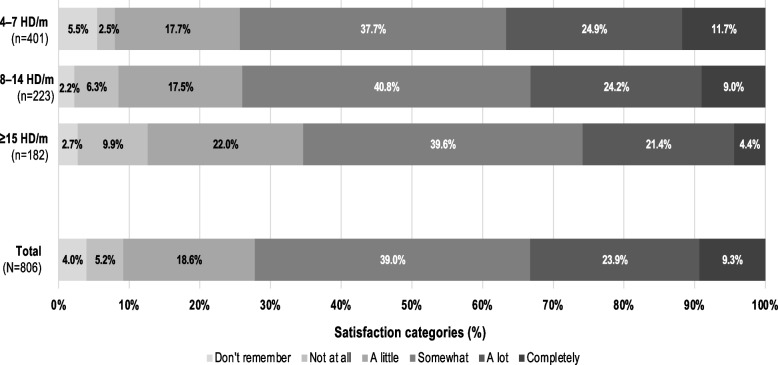
Patient satisfaction with current treatment in survey respondents eligible^a^ for migraine preventive medication (excluding calcitonin gene-related peptide-monoclonal antibody users; *n* = 806). ^a^Patients with  ≥  4 HD/m on average over the last 90 days and  ≥  11 (MIDAS). Users of CGRP-monoclonal antibodies are excluded. CGRP, calcitonin gene-related peptide; HD/m, headache days per month; MIDAS, Migraine Disability Assessment Score

Only 33.2% of traditional preventive users reported ‘a lot’ or ‘complete’ satisfaction with their current medication (Fig. 5). The proportions of patients with high levels of satisfaction (‘a lot’ or ‘complete’) decreased as the number of HD/m increased across the preventive eligible subgroups (from 36.6% to 25.8%).

## Discussion

We have described the sociodemographic and migraine-related clinical characteristics of the OVERCOME (EU) study migraine cohort, traditional preventive (orals and onabotulinum toxin A) treatment patterns observed in the migraine cohort and the subgroup survey respondents eligible for migraine preventive medication at the point in time the cross-sectional survey was taken, and patient satisfaction with current treatment in the preventive eligible subgroup. Given the new tailored treatment options offered by preventive and acute medications for migraine, it is important to understand the current status of migraine healthcare delivery, as well as the disease burden on people with migraine, and why people with migraine do not seek professional help, even though medical advice and treatment is accessible in their country. This study was specifically designed to capture this information, in particular with regard to preventive medications, from individuals with migraine in the real-world, outside of clinical trials and niche clinic migraine populations, including respondents who sought professional medical care and those who did not. The survey questions covered a broad range of topics to try to understand the burden of migraine and specifically how individuals cope with migraine on their own without professional medical support, e.g., the numbers and kinds of medications they use, and their satisfaction with those treatments.

As expected within the OVERCOME (EU) migraine cohort, the number of comorbidities reported by the respondents, the levels of disability (as measured by MIDAS) and the interictal burden (as measured by MIBS-4) all increased within increasing numbers of HD/m. It is striking that although approximately three-quarters of the migraine cohort sought professional medical care at some point in their lifetime, only half received a migraine diagnosis, and less than 20% of those who received a diagnosis had ever used preventive medication for migraine, indicating a lack of appropriate guideline-based care. In fact, 72.3% of all participants in the migraine cohort (including 63% of participants reporting ≥ 15 HD/m) had never taken preventive medication, regardless of whether or not they sought professional medical care and/or got a diagnosis. A broad range of reasons were cited by the survey respondents to explain this lack of preventive use, mostly driven by lack of or limited knowledge of the role of preventive medications in migraine and safety concerns.

Less than 15% of the OVERCOME (EU) survey respondents who received a migraine diagnosis were currently using preventive medication, and only one quarter of all participants in the migraine cohort were currently using preventive medication, regardless of whether or not they sought professional medical care and/or got a diagnosis. Even among those patients specifically eligible for preventive migraine medication (based on headache frequency and/or at least moderate disability due to migraine at the point in time the cross-sectional survey was taken), only one quarter were currently using preventives. These findings highlight the need to improve access to available preventive treatments, among those patients who are eligible and experiencing frequent and/or disabling migraine.

Of note, approximately 1 in 10 respondents currently using preventive medication were using three or more classes of traditional preventives (orals and onabotulinum toxin A), likely due to the presence of comorbidities, as some respondents reported only 0–3 HD/m. These results suggest high levels of self-medication among those respondents currently using preventive medications. However, the migraine cohort also included a significant proportion of participants with severe disability, severe interictal burden and considerable physical and emotional limitations, as confirmed by the patient-reported outcome measures included in the survey. Moreover, it should be noted that the survey did not capture how severe participants’ migraines were when they started taking multiple preventive medications.

Real-world evidence, such as that provided by the online OVERCOME (EU) survey, can provide important insights on unmet medical needs of people with migraine that are more difficult to obtain from other sources. In addition to highlighting the challenges of people with migraine not seeking care, possibly still due to stigma surrounding migraine, not getting a diagnosis and/or not taking preventive medication (ever or currently), this study identified several other issues. Half of all participants who had used traditional preventive medication (excluding CGRP-mAbs) took their last preventive medication for only a short period of time (i.e., six months or less), with concerns about lack of efficacy or poor tolerability commonly cited as reasons for stopping traditional preventive medications. Furthermore, only one-third of respondents in the subgroup eligible for migraine preventive medication reported ‘a lot’ or ‘complete’ satisfaction with their current medication (excluding CGRP-mAbs), suggesting the need for new preventive therapies that enhance patient satisfaction and improve long-term adherence.

The OVERCOME (EU) study comes at an important time when novel preventive therapeutics are available, adding a European perspective to the findings of the OVERCOME (US) and (Japan) surveys [7–9], and the US CaMEO longitudinal internet-based study [5], as well as adding to the literature in Europe (e.g., Eurolight) [3]. However, the OVERCOME (EU) study has several important limitations. The online survey data are self-reported and are susceptible to recall, misinterpretation and prioritisation biases. Furthermore, panel participants may not be a fully representative sample of the general population per country as a smaller-than-representative number of people between 55 and 65 years of age were included, possibly being due to the online survey format and the level of familiarity of the older age group with current online technologies.

## Conclusions

The OVERCOME (EU) study highlighted several unmet needs regarding preventive medications for migraine in Germany and Spain. Despite moderate to severe impairment, many people with migraine did not seek professional medical care, and many of those who did seek care were not receiving a diagnosis of migraine or appropriate guideline-based care, including preventive medication, or discontinued preventive treatment early. Furthermore, most patients classified as eligible for preventive medication (based on headache frequency and/or at least moderate disability due to migraine at the point in time the cross-sectional survey was taken) reported not using traditional preventives (excluding CGRP-mAbs) and many of those who did were not satisfied with their current standard of care. These findings highlight the low proportion of people diagnosed with migraine despite a higher rate of consultation and suggest the need for better access to treatment for people with migraine and new preventive therapies with improved efficacy and safety profiles to improve adherence and patient satisfaction.

## Data Availability

The data sets generated during and/or analysed during the current study are not publicly available due to the need for patient data protection.

## Abbreviations

ASC-12: Allodynia Symptom Checklist-12
BMI: Body mass index
CaMEO: Chronic Migraine Epidemiology and Outcomes study
CGRP: Calcitonin gene-related peptide
COVID-19: Coronavirus disease 2019
EQ-5D-5L: EQ-5D-5 Levels
EU: Europe
GAD-7: Generalized Anxiety Disorder scale-7
HD/m: Headache days per month
HCP: Healthcare professional
HCRU: Healthcare resource utilisation
HRQoL: Health-related quality of life
ICHD-3: International Classification of Headache Disorders, 3^rd^ edition
IMPAC: Impact of Migraine on Partners and Adolescent Children scale
mAb: Monoclonal antibody
MIBS-4: Migraine Interictal Burden Scale-4
MIDAS: Migraine Disability Assessment
MSQ v2.1: Migraine-Specific Quality-of-Life Questionnaire version 2.1
OTC: Over-the-counter
OVERCOME (EU): ObserVational survey of the Epidemiology, tReatment and Care Of MigrainE (Europe)
PHQ-8: Patient Health Questionnaire-8
QoL: Quality of life
SD: Standard deviation
SR-MD: Self-reported medical diagnosis of migraine
US: United States
VAS: Visual analogue score
WPAI: Work Productivity and Activity Impairment questionnaire

## References

[1] Stovner LJ, Andree C (2010). Prevalence of headache in Europe: a review for the Eurolight project. J Headache Pain.

[2] Doane MJ, Gupta S, Vo P (2019). Associations between headache-free days and patient-reported outcomes among migraine patients: a cross-sectional analysis of survey data in Europe. Pain Ther.

[3] Katsarava Z, Mania M, Lampl C (2018). Poor medical care for people with migraine in Europe – evidence from the Eurolight study. J Headache Pain.

[4] Buse DC, Manack AN, Fanning KM (2012). Chronic migraine prevalence, disability, and sociodemographic factors: results from the American Migraine Prevalence and Prevention Study. Headache.

[5] Manack Adams A, Serrano D, Buse DC (2015). The impact of chronic migraine: the Chronic Migraine Epidemiology and Outcomes (CaMEO) study methods and baseline results. Cephalalgia.

[6] Andrée C, Stovner LJ, Steiner TJ (2011). The Eurolight project: the impact of primary headache disorders in Europe. Description of methods. J Headache Pain.

[7] Lipton RB, Nicholson RA, Reed ML (2021). Diagnosis, consultation, treatment, and impact of migraine in the US: results of the OVERCOME (US) study. Headache.

[8] Hirata K, Ueda K, Komori M (2021). Comprehensive population-based survey of migraine in Japan: results of the ObserVational Survey of the Epidemiology, tReatment, and Care Of MigrainE (OVERCOME [Japan]) study. Curr Med Res Opin.

[9] Matsumori Y, Ueda K, Komori M (2021). Burden of migraine in Japan: results of the ObserVational Survey of the Epidemiology, tReatment, and Care Of MigrainE (OVERCOME [Japan]) study. Neurol Ther.

[10] Headache Classification Committee of the International Headache Society (IHS) The International Classification of Headache Disorders, 3rd Edition. (2018) Cephalalgia; 38(1): 1–211. 10.1177/0333102417738202 29368949

[11] Lipton RB, Bigal ME, Ashina S (2008). Cutaneous Allodynia in the migraine population. Ann Neurol.

[12] Stewart WF, Lipton RB, Whyte J (1999). An international study to assess the reliability of the migraine Disability Assessment (MIDAS) score. Neurology.

[13] Stewart WF, Lipton RB, Dowson AJ, Sawyer J (2001). Development and testing of the Migraine Disability Assessment (MIDAS) questionnaire to assess headache-related disability. Neurology.

[14] Jhingran P, Osterhaus JT, Miller DW (1998). Development and validation of the Migraine-Specific Quality of Life Questionnaire. Headache.

[15] Jhingran P, Davis SM, LaVange LM (1998). MSQ: Migraine-specific quality-of-life questionnaire: further investigation of the factor structure. Pharmacoeconomics.

[16] Martin BC, Pathak DS, Sharfman MI (2000). Validity and reliability of the Migraine-Specific Quality of Life Questionnaire (MSQ Version 2.1). Headache J Head Face Pain.

[17] EuroQol Research Foundation. EQ-5D-5L User Guide, 2019. Available from: https://euroqol.org/publications/user-guides. Accessed May 2022.

[18] Reilly MC, Zbrozek AS, Dukes EM (1993). The validity and reproducibility of a work productivity and activity impairment instrument. Pharmacoeconomics.

[19] Lipton RB, Buse DC, Adams AM (2017). Family impact of migraine: development of the Impact of Migraine on Partners and Adolescent Children (IMPAC) scale. Headache.

[20] Buse DC, Rupnow MFT, Lipton RB (2009). Assessing and managing all aspects of migraine: migraine attacks, migraine-related functional impairment, common comorbidities, and quality of life. Mayo Clin Proc.

[21] Buse D, Bigal MB, Rupnow M (2007). Development and validation of the Migraine Interictal Burden Scale (MIBS): a self-administered instrument for measuring the burden of migraine between attacks. Neurology.

[22] Pfizer Inc. Instruction Manual. Instructions for Patient Health Questionnaire (PHQ) and GAD-7 Measures. Available at: https://phqscreeners.pfizer.edrupalgardens.com/sites/g/files/g10016261/f/201412/instructions.pdf. Accessed July 2022.

[23] Ailani J, Burch RC, Robbins MS (2021). The American Headache Society Consensus Statement: update on integrating new migraine treatments into clinical practice. Headache.

[24] Lipton RB, Serrano D, Nicholson RA et al (2013) Impact of NSAID and triptan use on developing chronic migraine: results from the American Migraine Prevalence and Prevention (AMPP) study headache: J Head Face Pain 53(10):1548–1563 10.1111/head.12201 23992516

[25] Rodríguez-Almagro D, Achalandabaso A, Rus A et al (2020) Validation of the Spanish version of the migraine disability assessment questionnaire (MIDAS) in university students with migraine. BMC Neurol 20(1):67 10.1186/s12883-020-01646-y PMC7038557 32093620

[26] Benz T, Lehmann S, Gantenbein AR et al (2018) Translation cross-cultural adaptation and reliability of the German version of the migraine disability assessment (MIDAS) questionnaire. Health Qual Life Outcomes 16(1):42 10.1186/s12955-018-0871-5 PMC5845367 29523138

