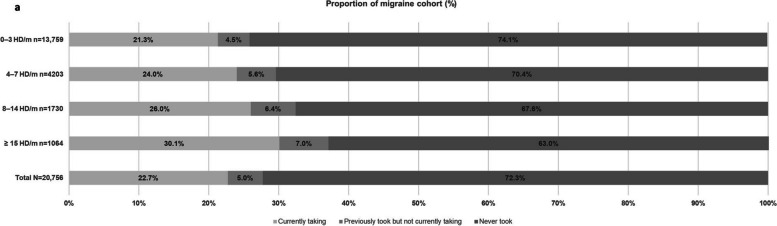# Correction: Preventive treatment patterns and treatment satisfaction in migraine: results of the OVERCOME (EU) study

**DOI:** 10.1186/s10194-023-01679-x

**Published:** 2023-10-20

**Authors:** Julio Pascual, Tommaso Panni, Grazia Dell’Agnello, Saygin Gonderten, Diego Novick, Stefan Evers

**Affiliations:** 1 Hospital Universitario Marqués de Valdecilla, Universidad de Cantabria and IDVAL, Santander, Spain; 2 Eli Lilly Deutschland GmbH, Bad Homburg, Germany; 3 Eli Lilly Italia SpA, Sesto Fiorentino, Italy; 4 Eli Lilly and Company Ltd, Dubai, UAE; 5 Eli Lilly and Company Ltd, Bracknell, UK; 6 University of Münster, Münster, Germany; 7 Department of Neurology, Lindenbrunn Hospital, Coppenbrügge, Germany

**Correction: J Headache Pain 24, 88 (2023)**

**https://doi.org/10.1186/s10194-023-01623-z**

Following publication of this article [1], the author group became aware of a typographical error in Fig. 4a which resulted in an incorrect representation of the data in the Figure.

The error caused the representation of the proportion of the total migraine cohort who never took preventive medication to be shown as 22.7%, instead of the correct proportion of 72.3%.

This error does not change the direction or significance of the results, interpretations, and conclusions of the manuscript.

The correct figure should have appeared as shown below and the original article has been corrected.

4a